# Antisense oligonucleotides: a novel Frontier in pharmacological strategy

**DOI:** 10.3389/fphar.2023.1304342

**Published:** 2023-11-17

**Authors:** D. Collotta, I. Bertocchi, E. Chiapello, M. Collino

**Affiliations:** ^1^ Department of Neuroscience Rita Levi Montalcini, University of Turin, Turin, Italy; ^2^ Neuroscience Institute Cavalieri Ottolenghi (NICO), University of Turin, Turin, Italy

**Keywords:** antisense oligonucleotide, siRNA, genetic disorder, gene silencing, pharmacology

## Abstract

Antisense oligonucleotides (ASOs) are short single stranded synthetic RNA or DNA molecules, whereas double-stranded RNA nucleotide sequences are called small interfering RNA (siRNA). ASOs bind to complementary nucleic acid sequences impacting the associated functions of the targeted nucleic acids. They represent an emerging class of drugs that, through a revolutionary mechanism of action, aim to directly regulate disease-causing genes and their variants, providing an alternative tool to traditional “protein-specific” therapies. The majority of the ASOs are designed to treat orphan genetic disorders that in most of the cases are seriously disabling and still lacking an adequate therapy. In order to translate ASOs into clinical success, constant technological advances have been instrumental in overcoming several pharmacological, toxicological and formulation limitations. Accordingly, chemical structures have been recently implemented and new bio-conjugation and nanocarriers formulation strategies explored. The aim of this work is to offer an overview of the antisense technology with a comparative analysis of the oligonucleotides approved by the Food and Drug Administration (FDA) and the European Medicines Agency (EMA).

## 1 Introduction

Last century has witnessed the drug development process primarily based on the small molecules and antibodies (Bennett, 2019). Recently, antisense oligonucleotides (ASO)-based research has gained momentum due to sequence-specific targets it employs for the therapeutic development. In particular, they represent an opportunity for strategical intervention to target RNA encoding proteins that are difficult to reach with conventional therapy, or to selectively target non-coding RNAs acting as activators/silencers of gene expression and dysregulated endogenous microRNAs that can lead to various diseases (Zamecnik and Stephenson, 1978; Levin, 2019). Although the first preclinical use of synthetic oligonucleotides to modulate RNA function dates back to 1978 (Zamecnik and Stephenson, 1978), their production as drugs has required constant improvements in chemistry, genomics, pharmacology, delivery and formulation platforms to increase their efficacy, safety and biodistribution (Levin, 2019). Recently, after many years of slow progress, ASO research is accelerating, with a variety of clinical trials that have reached their decisive stage during the years 2016–2020 (Shen and Corey, 2018). Via the interaction with different targets and through different molecular mechanisms, ASOs exert important positive effects in reducing oxidative stress, which exerts a pivotal role in several conditions, such as cancer, Alzheimer’s disease, diabetes, cardiovascular and inflammatory disorders. To date, the Food and Drug Administration (FDA) has approved thirteen ASOs for clinical use, while the European Medicines Agency (EMA) has authorised eight of them. In addition, several antisense drugs are now undergoing clinical trials for the management of cardiovascular, metabolic, endocrine, neurological, inflammatory and infectious diseases (Dhuri et al., 2020).

## 2 Mechanism of action of DNA and RNA antisense oligonucleotides

ASOs are polymers consisting of 15–21 nucleotides (Vegeto et al., 2019). There are different kinds of ASOs: single stranded DNA ASOs or RNA nucleotide sequences which are usually complementary to an endogenous miRNA (7), and double-stranded complexes, called short interfering RNA (siRNA) (Bajan and Hutvagner, 2020). ASOs are chemically synthesized to bind via complementary base-pairing a specific sequence of nucleic acid, as a messenger RNA (mRNA), or its nuclear precursor (pre-mRNA) (Levin, 2019).

When single stranded, ASOs have to be stable and able to be selectively addressed to their target before the degradation due to circulating nucleases. On the other hand, the duplex structure of siRNAs makes them more stable than ASOs in the blood as well as within the cells. However, systemic delivery of double-stranded siRNA is more challenging as they have larger molecular weight and negative charge. Moreover, they have hydrophilic phosphates on their outside surface, while the aromatic nucleobases are located mainly inside the duplex, thus leading to scarce interactions with plasma membranes and quite rapid renal excretion (Meister et al., 2004).

Once the ASOs has bound to the target RNA, a number of possible molecular mechanisms are triggered, which can be broadly classified as: (a) those promoting RNA cleavage through the recruitment of endogenous enzymes; (b) those that interfere with mRNA translation or maturation without promoting the degradation of the target (Ward et al., 2014). ASOs and siRNA are distinct classes of nucleic acid-based therapeutic molecules, each characterized by its own distinct mechanism of action. However, despite their individual disparities, both ASOs and siRNA possess remarkable potential as therapeutic agents, providing versatile means to modulate gene expression. This shared capacity makes them invaluable tools for addressing genetic diseases and holds the promise of reshaping disease progression through precise and targeted interventions. By collectively exploring ASOs and siRNA within the context of gene regulation strategies, we try to encompass the comprehensive landscape of nucleic acid-based therapeutics, acknowledging their distinctiveness while embracing their mutual contributions to advancing the field.

### 2.1 Cleavage of target RNAs by recruitment of endogenous enzymes

#### 2.1.1 RNA degradation by activation of ribonucleases (RNase)

One of the common mechanisms for RNA target inactivation is the induction of RNase H, a family of endonucleases that selectively cleave the RNA strand of RNA/DNA hybrids (Bennett and Swayze, 2010). In the context of mRNA/ASO heteroduplexes, RNase H induces the degradation of the target mRNA.

#### 2.1.2 RNA interference (RNAi)

siRNAs act by using the RNA interference (RNAi) pathway, a natural cellular defence mechanism evolved to recognize and degrade pathogenic RNAs (Bajan and Hutvagner, 2020). For this reason, siRNA intracellular protein association and binding to the target is generally highly selective and specific, making them suitable therapeutic tools (Schwarz et al., 2006).

siRNAs enter the physiological process of RNAi by associating with TAR RNA-binding protein (TRBP) and the argonaut 2 enzyme (AGO2), composing the RNA-Induced Silencing Complex (RISC). Once the siRNA is part of RISC, one RNA strand is cleaved and released, whereas the remaining guide strand finds the complementary mRNA, forming an RNA-RNA hybrid that induces AGO2 to degrade the target RNA (Meister et al., 2004).

The formation of RISC participates in cell-based defence, for example, against viruses. Despite that, siRNA-based drug applications are not limited to infective diseases, but they can be used for treating different types of cancers and various pathological conditions, including inflammatory disorders and neuropathies.

When the guide or sense strand binds off-target transcripts, the RNAi-related mechanism of action of siRNA may cause unwanted activity. An important safety concern related to therapeutic siRNA mechanism of action is the possible non-specific suppression of non-target mRNAs by the passenger strand. Also ASOs can induce off-target effects by association with sequences having a high degree of homology or by interactions with proteins (Chi et al., 2017).

### 2.2 Inactivation without mRNA degradation

Another mechanism used by ASOs for RNA inactivation is by preventing the interaction of mRNA with the ribosomes for reasons of steric hindrance. ASOs also interfere with mRNA maturation by constraining splicing or destabilizing nuclear pre-mRNA.

#### 2.2.1 Translation or maturation blockade of messenger RNA by steric hindrance

Due to steric bulk, after pairing with the mRNA, an ASO can block the translation of the transcript with one of the following mechanisms: i) by hindering its contact with the ribosomal 40S subunit; ii) by avoiding the assemblage of the 40S/60S subunits; iii) by hampering the sliding of the ribosome along the transcript; iv) by impeding the interaction with sequences that are essential for the maturation of the transcript, such as the addition of a 7-methylguanosine cap at the 5′ end and polyadenylation at the 3′ end (Bennett and Swayze, 2010). Steric hindrance can have a crucial role also in splicing modulation, as described below.

#### 2.2.2 Splicing modulation

**FIGURE 1:**
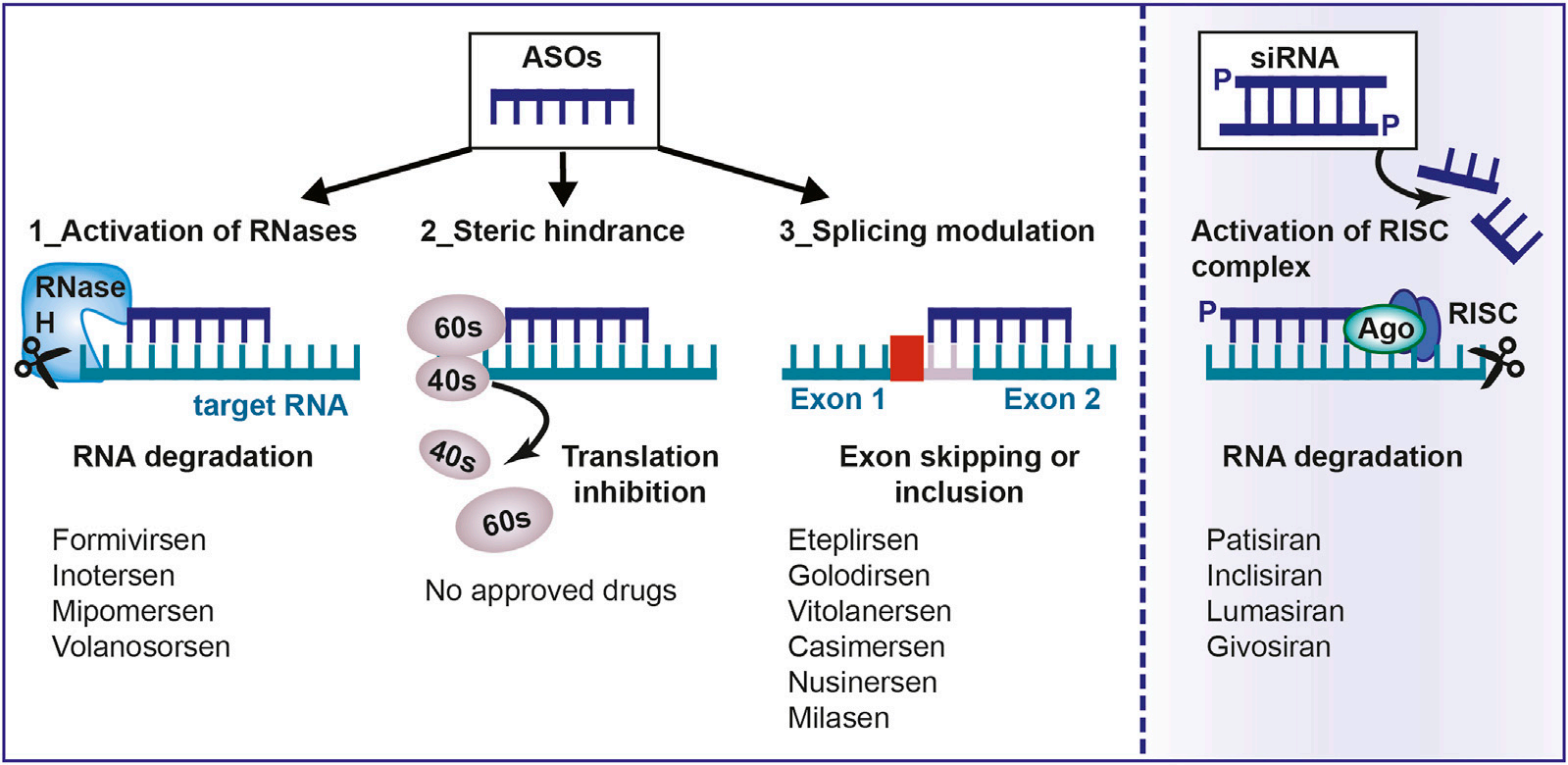
Mechanism of action of antisense oligonucleotides. Antisense oligonucleotides and siRNA can be used to target a specific, complementary (coding or non-coding) RNA. Main mechanisms of actions are: 1) RNA cleavage through the recruitment of endogenous enzymes; 2) Steric hindrance; 3) Splicing modulation; 4) Activation of RISC complex by the double-stranded siRNA. ASO, antisense oligonucleotides; aODN, siRNA: small-interfering RNA; RNase H, Ribonuclease H; RISC, RNA inducing silencing complex; mRNA, messenger RNA.

The goal of ASO therapy is to block the production of an abnormal form of protein or to restore the production of a protein that is lacking or not functioning (Havens et al., 2013). ASOs can bind the primary transcript within the nucleus and interfere with the spliceosome-mediated maturation process (Figure 1). Modulation of splicing by oligonucleotides consists of leading to exon skipping or exon inclusion. In exon skipping, ASOs bind pre-mRNAs and correct the mutation that caused the disruption of the reading frame by forming a transcript that, even if truncated, still encodes for a partially functional protein (Dhuri et al., 2020). In exon inclusion, ASOs are designed in order to target and repress regulatory elements resulting in the prevention of splicing events and the production of a full-length protein. In this regard, it is noteworthy to mention that not only linear motifs, but also RNA secondary structures that are formed because of long-distance interaction (LDI), regulate alternative splicing and can be targeted by ASOs. One of the first report about this mechanism is the study by Singh et al., 2013 who, by using cells from patients affected by spinal muscular atrophy (SMA), demonstrated the effectiveness of an ASO in correcting SMN2 exon 7 splicing, via the sequestration of the 3′ strand of an unique intra-intronic structure termed internal stem through LDI-1 (ISTL1) (Singh et al., 2013).

#### 2.2.3 miRNA inhibition

MicroRNAs (miRNA) are small RNA molecules essential for gene regulation. They bind AGO proteins forming RISC, sharing the same proteins used by RNAi. Indeed, given that siRNA drugs compete with endogenous miRNAs to bind AGO2, it is important to optimize their dose to avoid RISC saturation and, in turn, inhibition of the RISC complex.

### 2.3 Aptamers

A particular class of single-stranded DNA or RNA oligonucleotides is represented by aptamers. They have a three-dimensional structure that allows a highly specific interaction with proteins (Wiraja et al., 2014), this means that aptamer binding is mainly determined by its tertiary structure and not by the primary sequence. Unlike the previous classes, aptamers are identified through the Systematic Evolution of Ligands by Exponential Enrichment (SELEX) (Ohuchi, 2012). An aptamer currently in clinical use is pegaptanib, an RNA aptamer directed against vascular endothelial growth factor (VEGF)-165, responsible for pathological ocular neovascularization associated with age-related macular degeneration (Ng et al., 2006). Another aptamer that has been recently approved for the treatment of geographic atrophy, also secondary to age-related macular degeneration, is avacincaptad pegol, an inhibitor of the complement component 5 (Kang, 2023).

## 3 Pharmacokinetics

Having explored the diverse mechanisms by which ASOs exert their effects on target RNA, it is crucial to delve into their pharmacokinetics to better understand how these therapeutic molecules behave within the body. Understanding of pharmacokinetics is important in the drug development and safety field. This is particularly true for ASOs, where pharmacokinetics is intricately intertwined with the chemical properties of these therapeutic molecules. ASOs undergo various chemical modifications that impact their internucleotide links (first generation ASOs), the connecting rings between the ASO backbone and nucleobases (second generation ASOs), and the nucleobases themselves, which play a vital role in complementary base pairing with target RNA. These chemical alterations not only define the structural characteristics of ASOs but also govern their pharmacokinetic behavior, ultimately influencing their therapeutic effectiveness and safety profiles (Shadid et al., 2021).

The main route of administration for ASOs is the parenteral one (either intravenous or subcutaneous) (Geary et al., 2015). After subcutaneous (SC) infusion, ASOs move into the circulation, with peak concentrations reached within 3–4 h for the second generation of ASOs (Levin et al., 2007). Plasma concentration decrease in a multi-exponential mode characterised by a dominant rapid first phase of distribution in which the drug is transported to tissues (minutes/hours) (Geary et al., 2015), followed by a slower second phase of elimination (the half-life can reach up to several weeks for ASOs with both backbone and sugar modifications). The first phase of distribution is largely driven by backbone chemistry (Levin et al., 2007). The phosphorothioate (PS) backbone gives an important pharmacokinetic benefit, given that the increased binding to proteins allows a significant release to many tissues. Indeed, tissue distribution of PS-modified ASOs is relatively broad, with the organs reaching the highest concentration being the liver and kidney, followed by bone marrow, adipocytes and lymph nodes (Vegeto et al., 2019). At the tissue level, intracellular uptake of ASOs occurs by endocytosis mediated by surface receptors (integrins, scavenger receptors and Toll-like receptors) (Vegeto et al., 2019). Passive diffusion of ASOs into cells is limited by the fact that they are large molecules (single-stranded ASOs are 4–10 kDa, double-stranded siRNA are 14 kDa) and have a negatively charged surface because of phosphate groups, resulting in electrostatic repulsion with the cell membrane (Roberts et al., 2020). Systemically delivered drugs directed at the central nervous system (CNS) encounter a further impediment to their action, namely, the blood-brain barrier (Bennett et al., 2017).

Subsequent to their release from the endolysosome, ASOs navigate to their designated site of action, endeavoring to evade degradation or re-exportation via exocytosis (Sahay et al., 2013). Following their liberation from the endolysosome, these ASOs exhibit extended half-lives (ranging from 2 to 4 weeks) and exert prolonged inhibitory effects on the expression of their target RNA (Geary et al., 2015). The subsequent elimination phase operates through a diverse array of intricate mechanisms, collectively ensuring the methodical clearance of ASOs from the biological system. Among these mechanisms, direct excretion via the renal glomerular filtration system emerges as a crucial avenue, facilitated by the kidneys’ finely tuned filtration capabilities. Concurrently, ubiquitous tissue and serum endo- and exonucleases engage in a metabolic interplay, progressively degrading ASOs and priming them for subsequent elimination. The orchestrated choreography extends to circulating macrophages, vigilant custodians of the endothelial reticulum system, engaging in the meticulous task of phagocytic uptake. These sentinel cells envelop the ASOs, facilitating their eventual removal from circulation. Additionally, specific plasma proteins contribute to the orchestration of this elimination phase, potentially inactivating or sequestering ASOs and guiding their transition from active therapeutic agents to inert remnants (Kilanowska and Studzińska, 2020). Currently, all approved ASOs are either administered locally, such as directly into the eye and the cerebrospinal fluid, or act in the liver after parenteral administration (Roberts et al., 2020). Intravitreal administration limits systemic absorption to 1% of the administered dose, minimising the toxicological risk (Bennett et al., 2017). This route has been used for the delivery of fomivirsen, to treat cytomegalovirus retinitis. Intrathecal administration is required to treat diseases of the CNS. It ensures a high distribution in the cortical tissue and spinal cord and a lower concentration in deeper brain structures (Geary et al., 2015). Lastly, drug delivery to the liver by parenteral administration is facilitated by several factors: the high perfusion of the liver, the presence of a fenestrated sinusoidal endothelium that allows the passage of ASOs and the high concentration on the hepatocyte membrane of receptors that can facilitate the endocytotic process (e.g., the asialoglycoprotein receptor, ASGR) (Tanowitz et al., 2017). In contrast, the development of technologies for extra hepatic delivery is still the main goal in the field of ASOs.

## 4 Strategy to enhance delivery

### 4.1 Chemical modification

The use of chemical modifications has been crucial to increase ASO enzymatic stability, target recognition, binding efficiency, tissue distribution and, at the same time, to reduce their toxicity, leading to significant improved antisense drugs (Edvard Smith and Zain, 2019). The most common and relevant modifications of currently marketed oligonucleotides are applied at the level of the backbone (first generation ASOs), the heterocycle and the sugar, as discussed below. The key role played by the introduced chemical components is demonstrated by the fact that most of the ASOs approved to date are “naked”, i.e., without an additional delivery vehicle (Roberts et al., 2020).

#### 4.1.1 Backbone modifications/phospodiester linkage modifications

The primary reason for modification of the oligonucleotide backbone is the inherent instability of the phosphodiester bond to nucleases. This can be prevented by the non-bridging replacement of the phosphodiester bond with a sulphur atom (Eckstein, 2000). The phosphorothioate (PS) backbone confers a delay in renal clearance by increasing both resistance to nucleases action and binding to plasma (e.g., albumin) and intracellular (e.g., nucleolin) proteins (Gaus et al., 2019). PS ASOs, (first generation), are stable in plasma and tissues (half-life: 2–3 days). PS binding allows the activation of RNase H. Although it creates a minor reduction in binding affinity to the target, it is possible balance it by some modifications, such as, e.g., additional modifications to the sugar backbone portions (Roberts et al., 2020; Oberemok et al., 2018). Moreover, PS ASOs enter cells more easily: PS is recognised by scavenger receptors (such as STAB1 and STAB2 stabilins) that facilitate internalisation in tissues such as the liver (Miller et al., 2018). Despite this, *in vivo* studies have demonstrated the necessity of high and repeated doses to achieve the therapeutic effect (Vegeto et al., 2019). A toxicological limitation of PS ASOs is the triggering of immune reactions by activation of Toll-like receptors (TLRs) (Vegeto et al., 2019). Moreover, an extra sulfur atom can be added into the PS linkage, resulting in a chiral centre at each modified phosphorous atom, producing two possible stereoisomeric forms having different physiochemical properties and pharmacological activity (Roberts et al., 2020).

Besides the modifications of the phosphorodextrin backbone linkage, isosteric substitutions of the phosphoriboside group have been implemented giving rise to two classes of ASO analogues: phosphorodiamidate morpholino oligomers (PMOs) and nucleic peptide acids (PNAs). PMOs are characterized by the substitution of ribose with morpholino and of the phosphorodextere bond with a phosphorodiamine bond. PNAs, on the other hand, are synthetic nucleic acid imitations containing neutral N-2-aminoethyl glycine units, with nucleobases linked by a flexible methyl carbonyl linker (Dhuri et al., 2020). Both classes are neutral and therefore form very stable hybrids with the target RNA and have high resistance to nuclease action (Bennett et al., 2017). However, they do not support the activity of endogenous enzymes such as RNAse H or AGO2 and exert their action through mechanisms of steric hindrance and splicing modulation (Bennett et al., 2017). The lack of binding to serum proteins results in a rapid renal clearance.

#### 4.1.2 Heterocycle/nucleobase modifications

The main positive effect of modifications applied to the heterocycle is the increase of the binding affinity to the target (Herdewijn, 2000). One of the most implemented adjustment is the replacement of the C5 hydrogen of deoxycytidine with a methyl group (Vasquez et al., 2021). The introduction of the methyl group between the nitrogenous bases in the major groove confers increased thermal stability to the duplex (Sahay et al., 2013). This modification supports RNase H activity and is used to reduce the immunostimulatory potential of PS ASOs (Bennett et al., 2017).

#### 4.1.3 Ribose sugar modifications

Currently, modifications of the hydroxyl in the 2′ position of the sugar portion of deoxyribose in DNA and ribose in RNA, respectively, have made the greatest contribution in increasing the pharmacological properties of oligonucleotides by creating second-generation ASOs (Bennett et al., 2017). The most commonly used substitutions are the fluorine group (2′-F), the methyl group (2′-O-Me) and the methoxymethyl group (2′-O-MOE) (Tanowitz et al., 2017). This variation is notable, on one hand, for its increased nuclease resistance, as it blocks the nucleophilic portion of the hydroxyl at the 2′ position of the sugar, and, on the other hand, for its increased thermal stability at complementary hybridisation, which allows tighter binding and the use of shorter oligonucleotides (Shen and Corey, 2018). Unfortunately, modifications at the 2′ position are not compatible with RNAse H-mediated cleavage activity (Shen and Corey, 2018). This limitation was minimised through the use of the gapmers strategy. This method makes use of a central region of unmodified DNA or RNA ASOs supporting RNase H activity flanked by ends of 20 nucleotides modified in 2' (Tanowitz et al., 2017). For siRNA, the situation is more complex as they must retain the ability to be recognised by the AGO2 cleavage enzyme. However, the RNAi machinery is remarkably tolerant to chemical modifications carried out at the 2′ position of the sugar (e.g., givosiran) (Roberts et al., 2020; Allerson et al., 2005).

Instead Bridged nucleic acids (BNAs) contain a constrained bridge between 2′ oxygen and 4’ carbon of the ribose ring (Dhuri et al., 2020). These bicyclic systems have been fused into the flanking regions of gapmers as they have an ideal conformation to interact with complementary DNA or RNA sequences showing a strong increase in binding affinity (Roberts et al., 2020).

#### 4.1.4 Terminal modification

Phosphorylation at the 5′ end of the siRNA guide strand is crucial for activity, because it mediates the binding to AGO2 (Schirle and MacRae, 2012; Frank et al., 2010). To limit the subtraction of this group by cellular phosphatases, the introduction of 5′-vinyl phosphonate as an imitation phosphate that is not a substrate of phosphatases has been functionally introduced (Haraszti et al., 2017). Terminal modifications can drastically improve the therapeutic efficacy of small ASOs. For instance, the incorporation of PEG-282 and propyl modifications at the 5′ and 3′ ends, respectively, of a small ASO (8-mer), ameliorated its effectiveness *in vivo*, leading to improved symptoms in both severe and mild mouse models of spinal muscular atrophy (SMA) (Keil et al., 2014).

### 4.2 Nanoformulation-based delivery

Advances in nanotechnology make it possible to overcome certain pharmacokinetic limitations of ASOs, including plasma and endosomal degradation, direct renal clearance and intracellular transmembrane delivery (Roberts et al., 2020). The nanoparticles, absorbed by endocytosis, are, however, heterogeneously sized (around 100 nm) with limited biodistribution due to difficulty of diffusion through the extracellular matrix of tissues (Tanowitz et al., 2017). Their action is mainly concentrated in the liver and in the reticuloendothelial system, since the sinusoidal capillary endothelium has sufficient openings to allow nanocarriers in (Wisse et al., 2008). Nanocarriers are also a potential approach in the field of oncology, as they show an accumulation in the tumour environment due to the EPR (enhanced permeability and retention) effect (Dhuri et al., 2020).

#### 4.2.1 Lipoplexes and liposomes

Lipid-based delivery systems, such as lipoplexes and liposomes, derive from mixing polyanionic oligonucleotides with lipids. These formulations are able to mask the negative charge of the ASOs in order to reduce electrostatic repulsion with the cell surface (Vegeto et al., 2019). Specifically, polyplexes result from the direct interaction between polyanionic ASOs and polycationic lipids (Rehman et al., 2013). In contrast, liposomes are closed and stable vesicular systems characterised by a lipid bilayer in which the ASO is confined within the aqueous space (Ickenstein and Garidel, 2019). Some lipid nanoparticles (LNPs, stable nucleic acid lipid particles) consist in polycationic lipids (Roberts et al., 2020). In order to minimise the interaction with plasma opsonins, which lead to rapid degradation by phagocytes of the reticuloendothelial system, a shielding strategy has been adopted. The most commonly used polymer to create a superficial steric barrier to shield the residual charge is polyethylene glycol (PEG) (Ambegia et al., 2005). An example of LNP-based siRNA formulation is Patisiran used to treat transthyretin-mediated amyloidosis.

#### 4.2.2 Polymeric nanoparticles

Polymeric nanoparticles result from the non-covalent complexation between negatively charged ASOs and cationic polymers, including PEI (polyethylenimine), PBAE (poly-(beta-amino ester)) or poly-L-lysine (Geary et al., 2002). After endocytosis, in order to transport the ASO from the endolysosome to the cytoplasmic compartment, PEI and PBAE have an effect known as the “proton-sponge effect” (Benjaminsen et al., 2013), whereas poly-L-lysine-based nanoparticles incorporate fusogenic peptides or lytic domains capable of destabilising the endosomal membrane (Geary et al., 2002). However, the clinical advancement of polymeric nanoparticles has been limited by bioincompatibility and the inevitable toxicity in chronic use resulting from the undesired interaction between the chaperone charge and serum and tissue proteins (Dhuri et al., 2020).

#### 4.2.3 Exosomes

Exosomes are biological nanoparticles whose use as delivery systems is current under study at a preclinical level. In particular, exosomes are extracellular bilayer vesicles, ranging in size from 30 to 100 nm, whose function is to facilitate intercellular communication through the transfer of nucleic acids, lipids and proteins (Tanowitz et al., 2017). Exosomes show many benefits related to oligonucleotide drug delivery as they are able to cross biological membranes, are protected from phagocytosis and are not toxic (Roberts et al., 2020).

#### 4.2.4 Spherical nucleic acid (SNA)

SNA particles consist of a densely packed shell of nucleic acids, which are oriented around a hollow or solid core nanoparticle. SNA particles are currently undergoing clinical trials for the administration of ASOs to tumors (such as glioblastoma) or for their topical administration in the treatment of psoriasis (Kapadia et al., 2018).

### 4.3 Conjugation strategies

The potential delivery of ASOs to DNA and RNA can be enhanced by conjugation with ligands able to promote cellular uptake and active targeting by interaction with superficial receptors and, on the other hand, to reduce the direct renal clearance of ASOs by increasing their size (Roberts et al., 2020). The most commonly used ligands are lipids, sugars, peptides, aptamers or antibodies. Unlike nanoparticles, they are well-defined molecular entities characterised by standard techniques, with a favourable biodistribution profile (Tanowitz et al., 2017).

#### 4.3.1 Lipid conjugates

The main result of this technological approach concern exposure to the liver, that have been achieved through conjugation with cholesterol and lipid groups such as long-chain fatty acids and α-tocopherol (Bennett et al., 2017). It has been shown that the *in vivo* activity of lipid-conjugated ASOs depends on their ability to bind plasma lipoproteins and thus exploit the endogenous system for lipid transport and uptake (Roberts et al., 2020).

#### 4.3.2 GalNac conjugates

An optimal delivery strategy to hepatocytes is trivalent conjugation of N-acetyl-galactosamine (GalNac) to the siRNA passenger strand or single-stranded DNA ASOs (Prakash et al., 2014). This ligand is a carbohydrate portion with high affinity and selectivity for asialoglycoprotein receptor 1 (ASGR1), which is densely expressed at the plasma membrane of hepatocytes (approximately 5*105 copies per hepatocyte) (Tanowitz et al., 2017). Upon ligand binding, the receptor-ligand pair is internalised in the endosomes. The acidic endomembrane environment promotes dissociation of the ligand from the receptor, allowing the receptor to coming back to the plasma membrane and the ligand-conjugate to be available for pharmacological action (Springer and Dowdy, 2018). Subsequently, the GalNac portion undergoes enzymatic degradation. Conjugation with GalNac increases the power of the ASO by approximately 30-fold and increases entry into hepatocytes (otherwise unconjugated ASOs have been detected mainly in non-parenchymal liver cells) (Roberts et al., 2020). This evidence results in an increase in the therapeutic index and, consequently, in a lower dose and/or a lower frequency of administration. In clinic, there are two examples of GalNac-conjugated siRNA: Givosiran, monthly administered to treat acute hepatic porphyria; and Inclisiran, administered twice a year in patients with familial hypercholesterolaemia. However, these important results are partly due to the liver being one of the main tissues of oligonucleotide accumulation and partly to ASGR1 receptor having many desirable features such as high expression levels, rapid internalisation and recycling (turnover of about 20 min) (Tanowitz et al., 2017).

#### 4.3.3 Peptide conjugates

Cell-penetrating peptides (CPPs) are short peptide sequences with positive charge that have been identified as carrying neutrally charged ASOs, with which they are linked, such as PMO and PNA, across cell membranes. CPPs can penetrate into the cell through endocytosis or by perturbing the plasma membrane lipid bilayer (Tréhin et al., 2004). The use of ASO conjugated with peptides is also found in the CRISPR-Cas9 system. CRISPR-Cas9 is composed of a nonspecific nuclease (Cas9) and a series of programmable sequence-specific CRISPR RNA (crRNA), which lead to DNA cleavage by Cas9 and produce double-strand breaks once the target sites are identified (Karimian et al., 2019).

#### 4.3.4 Antibodies and aptamer conjugates

Even though a number of technologies for hepatic delivery are available, extrahepatic targeting is still a challenge (Bennett et al., 2017). Currently, antibody- and aptamer-conjugated ASOs are in the early stages of testing in order to exploit their interaction with specific superficial receptors (e.g., transferrin receptor highly expressed in skeletal and cardiac muscle) (Roberts et al., 2020). Aptamers have a number of advantages over antibodies, including facilitating the production by chemical synthesis, low immunogenicity and lower cost (Vegeto et al., 2019).

## 5 Toxicology

The most toxicologically characterised classes of ASOs are the first-generation ones (ASO-PS) and the second-generation ASOs with 2′-O-MOE sugar adjustments due to the larger number of drugs in clinical use. The toxicity of ASOs is dose-proportional and can be affected by both the oligonucleotide and the formulation (Bennett et al., 2017).

### 5.1 Toxicity linked to the oligonucleotide

The toxicity of ASOs is commonly categorised into: hybridization-dependent or hybridization-independent way (Frazier, 2015).

#### 5.1.1 Hybridization-dependent

Hybridization-dependent toxicity includes both excessive pharmacological effects that occur between the ASO and the target; and non-specific effects due to complementary or partial recognition of unwanted transcripts (Batista-Duharte et al., 2020).

Off-target toxicity can be avoided or minimised by implementing bioinformatics analyses for target RNA selection and sequence homology detection, performing accurate characterisation of pharmacology and toxicology in preclinical models and implementing chemical modifications (Bennett et al., 2017). A recent study conducted by Scharner et al. shows the *in vitro* effects of splice-modulating ASOs on 108 potential off-targets predicted on the basis of sequence complementarity, and identified 17 mis-splicing events for one of the ASOs tested (Scharner et al., 2020). Based on analysis of data from two overlapping ASO sequences, they conclude that off-target effects are difficult to predict, and the choice of ASO chemistry influences the extent of off-target activity. The off-target events caused by the uniformly modified ASOs tested in their study were significantly reduced with mixed-chemistry ASOs of the same sequence. Furthermore, using shorter ASOs, combining two ASOs and delivering ASOs by free uptake also reduced off-target activity. Finally, ASOs with strategically placed mismatches can be used to reduce unwanted off-target splicing events.

A more practical example that highlights the importance of implementing better ASO design is the results obtained by Ottesen et al. (2021). In this study, the transcriptome of SMA patient cells treated with 100 nM of Anti-N1 for 30 h was analyzed. While 100 nM of Anti-N1 substantially stimulated SMN2 exon 7 inclusion, it also caused massive perturbations in the transcriptome and triggered widespread aberrant splicing, affecting expression of essential genes associated with multiple cellular processes. The authors also showed a substantial reduction in off-target effects with shorter ISS-N1-targeting ASOs.

At the clinical level, toxicity may be negligible as not all RNA sites are accessible, not all tissues have pharmacological ASO concentrations and gene silencing does not necessarily lead to toxicity.

#### 5.1.2 Hybridization-independent

There are four subcategories of non-pharmacological toxicity:

i. Tissue accumulation in the kidney and liver - The consequences related to the concentration of ASOs in the kidney and liver tissues are the most frequently encountered in preclinical toxicity studies through *in vivo* and *in vitro* studies (Frazier, 2015). They are related to the distribution and metabolism of ASOs. In the liver, ASO accumulation is mainly mediated by sinusoidal endothelial cells, followed by hepatocytes and Kupffer cells (Vegeto et al., 2019). In the kidney, accumulation of ASOs occurs at high doses by reabsorption in the cells of the proximal convoluted tubule following glomerular filtration. The main symptom of liver toxicity occurs with increased levels of circulating liver enzymes; in contrast, increased tubular proteinuria (rarely glomerular nephritis) due to disruption of tubular reabsorption capacities is the main indication of renal damage (Vegeto et al., 2019). Histological analysis of both liver and kidney tissues shows the presence of ASOs in basophilic granules at the level of the cytoplasm of epithelial cells related to an increased incidence of degenerative alterations especially in chronic use. A second common histological change is the increase in granular or vacuolated macrophages linked to cellular activation and the secretion of pro-inflammatory cytokines (Frazier, 2015).
ii. Aptamer binding - The interaction of the ASO in plasma, intracellular or cell surface proteins is known as the “aptamer effect.” A common consequence is the activation of complement, coagulation and immunity (Bennett and Swayze, 2010). Generally, this toxicity is influenced by the oligonucleotide chemical class and does not depend on the sequence (Geary et al., 2015). An exception is the activation of the Toll-like receptor of innate immunity, which has shown to be dependent on sequences rich in guanine and uracil at the 3′ end (Krieg, 2006). This immunomodulatory effect, however, can be easily minimised by avoiding these sequence motifs.
iii. Pro-inflammatory mechanisms - Reactions at the injection site, fever, chills and nuchal rigidity were commonly observed during clinical trials of phosphorothioate ASOs (e.g., mipomersen) (Levin, 2019).
iv. Thrombocytopenia - At the clinical level, thrombocytopenia is a side effect that may hamper the development of ASOs after the use of gapmer PS-2′-O-MOE (Sahay et al., 2013). For example, severe thrombocytopenia has been reported in patients with transthyretin-mediated hereditary amyloidosis treated with inotersen. In these patients, monitoring of platelet counts and, if necessary, adjustment of the medicine dose is required.

### 5.2 Adverse effects caused by formulation

Proinflammatory and immunostimulatory effects are linked to the residual positive surface charge of some lipid nanoparticles used to promote siRNA biodistribution. Therefore, lipid nanoparticle formulations, as in the case of patisiran, require premedication with antihistamines, glucocorticoids and non-steroidal anti-inflammatory medicines to avoid unwanted effects (Coelho et al., 2013).

### 5.3 ASOs as interesting tools against oxidative stress

Encouraging results obtained mainly in preclinical studies suggest ASOs as interesting tools against oxidative stress. In C57BL/6J mice, an ASO was demonstrated to modulate the activity of a phosphatidylethanolamine N-methyltransferase (PEMT), that positively regulates mitochondrial ubiquinone (CoQ) content. Acute PEMT disruption mediated by ASOs was sufficient to increase mitochondrial CoQ and decrease superoxide, resulting in the preservation of insulin sensitivity and an improvement of glucose and insulin responses in high fat diet (HFD)-fed mice (Ayer et al., 2021).

Moreover, in HFD-fed mice, the treatment with generation 2.5 ASOs, which are highly potent and contain 2′–4′ constrained ethyl (cEt)-modifications that allow the binding to RNase H1.20, were used against the mammalian STE20-like protein kinase 3 (MST3) and resulted in protection against diet-induced oxidative stress at hepatic level, improving the full spectrum of HFD-induced nonalcoholic fatty liver disease, including suppressed liver steatosis, inflammation, fibrosis, and cellular damage (Caputo et al., 2021). In particular, Mst3 ASOs was able to blunt lipogenic gene expression, and accumulation of acetyl-CoA carboxylase (ACC) protein leading to a reduction of the oxidative and ER stress induced by hepatic lipotoxicity in obese mice.

The same authors had previously reported that the knockdown of MST3 in cultured human hepatocytes stimulates β-oxidation and triacylglycerol (TAG) and inhibits fatty acid influx and lipid synthesis, instead MST3 overexpression increases lipid accumulation in mouse and human liver cells. This resulted in an increase in density and size of intracellular lipid droplets, which is known to cause cellular dysfunction and contribute to the development of lipid-related metabolic disorders. The authors in fact reported that reducing or increasing MST3 abundance in human cultured hepatocytes leads to a suppression or an aggravation of oxidative stress, respectively (Cansby et al., 2019).

In a murine model of Alzheimer disease (AD), a phosphorothionated antisense against glycogen synthase kinase (GSK)-3β, GAO, lead to an improvement of learning and memory abilities coupled with a reduction of protein oxidation and lipid peroxidation markers (Farr et al., 2014). This decrease in oxidative stress was associated to a concomitant increased levels of the antioxidant transcription factor nuclear factor-E2-related factor 2 (Nrf2), which is negatively regulated by GSK-3β.

Similarly, siRNA directed against another negative modulator of Nrf2 protected against oxidative stress *in vitro* and showed protective effects against MPTP-induced dopaminergic terminal damage *in vivo* when injected into the striatum (Williamson et al., 2012).

Other reports revealed suppressive effects of an ASO with an analogous structure as nusinersen on CNS oxidative stress and microglial activation, suggesting also a new role of SMN protein in microglia (Ando et al., 2020). In addition, in Spinal muscular atrophy (SMA) type 1 patients, preliminary results suggest that the therapy with nusinersen is indeed effective in reducing inflammation and oxidative stress (Bianchi et al., 2021). In fact, 6 months after starting treatment it was observed a reversion of the cerebrospinal fluid protein pattern from patient cohort to that of control donors. Remarkably, an upregulation of apolipoprotein A1 and E were detected. Since these multifunctional proteins are critically active in biomolecular processes aberrant in SMA, i.e., neuronal survival and plasticity, inflammation, and oxidative stress control, their nusinersen induced modulation may support SMN improved-expression effects.

Lastly, regarding miRNA inhibitors, an anti-miR-21 was demonstrated to be highly effective in murine models of Alport nephropathy: in particular, anti-miR-21 was able to reduce TGF-β-mediated stress response in glomeruli, to mitigate the expression of PPAR-α and its downstream fatty acid oxidation, to inhibit pro-inflammatory and profibrotic signals and to control the production of reactive oxygen species (Ben-Nun et al., 2020). Based on these evidences, clinical trials have been started with two miR-21 inhibitors, RG-012 and Lademirsen (SAR339375), in patients with kidney fibrosis as a result of Alport syndrome (Available from: https://clinicaltrials.gov/ct2/show/NCT03373786 and https://clinicaltrials.gov/ct2/show/NCT02855268).

## 6 FDA and EMA-approved formulations

**FIGURE 2:**
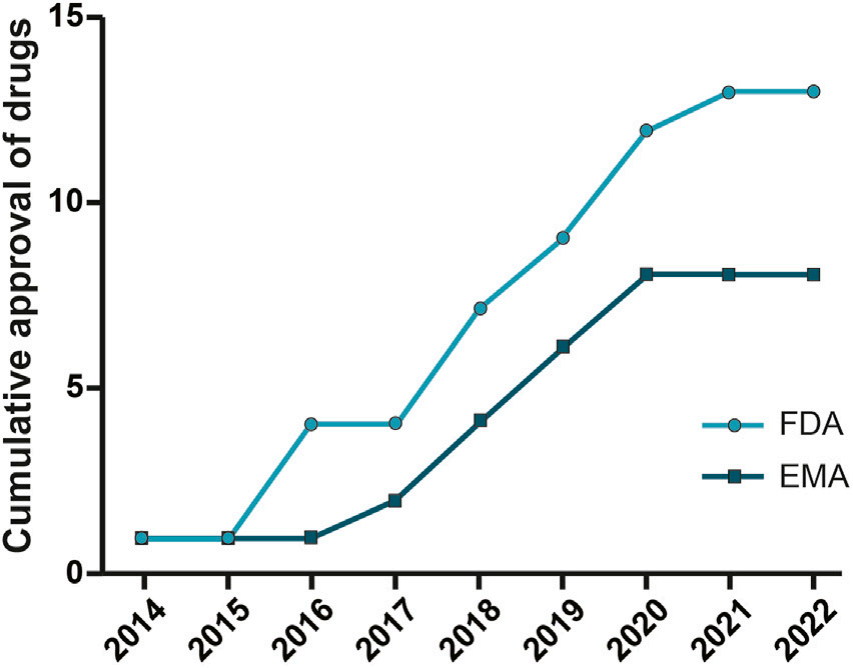
Oligonucleotide drugs approved by FDA and EMA. ASO drugs for different therapeutic areas (neurological and neuromuscular, metabolic and endocrine, and infection diseases) approved by the Food and Drug Administration and European Medicines Agency from 2013 to 2022.

As shown in Table 1 and Figure 2 there are already different ASOs approved from FDA and EMA for the treatment of several disorders, including metabolic/endocrine, neurological/neuromuscular, cardiovascular and infectious diseases.

**TABLE 1:** FDA and EMA approved antisense drugs.

Name (market name)Company	Chemistry	Mechanism of action	Target/organ	Indication	Route/Dosing	Year of approval	Designation
Fomivirsen (Vitravene™), Ionis Pharma, Novartis	21 mer PS DNA	RNase H1	CMV IE-2 mRNA/eye	CMV retinitis	IVT/300 µg every 4 weeks	FDA (1998)	-
						EMA (1999)	
Mipomersen (Kynamro™), Ionis Pharma, Genzyme, Kastle Tx	20 mer, 2′-O-MOE, PS, 5-methyl cytosine	RNase H1	Apo-B-100 mRNA/liver	HoFH	SC/200 mg once weekly	FDA (2013)	Orphan
Eteplirsen (Exondys 51^®^), Sarepta Tx	30 mer PMO	Exon skipping	DMD pre-mRNA exon 51/Skeletal muscle	DMD	I.V. infusion/30 mg/kg once weekly	FDA (2016)	Orphan
Nusinersen (Spinraza^®^), Ionis Pharma, Biogen	18 mer PS, 2′-O-MOE, 5-methyl cytosine	Exon inclusion	SMN2 pre-mRNA exon 7/CNS	SMA	ITH/12 mg once every 4 months	FDA (2016)	Orphan
						EMA (2017)	
Patisiran (Onpattro^®^), Alnylam	2′-O-Me, 2′F, PS siRNA	AGO2	TTR mRNA/liver	hATTR	I.V. infusion 0.3 mg/kg once every 3 weeks	FDA (2018)	Orphan
						EMA (2018)	
Inotersen (Tegsedi^®^), Ionis Pharma, Akcea Pharma	20 mer 2′-O-MOE, PS	RNase H1	TTR mRNA/liver	hATTR	SC/300 mg once weekly	FDA (2018)	Orphan
						EMA (2018)	
Vutrisiran/ALN-TTRSC02	2′-O-Me, 2′-F,PS	RNase H1	TTR mRNA/liver	hATTR	SC/25 mg every 3 months	FDA (2022)	Orphan
Milasen Boston Children’s Hospital	22 mer 2′-O-MOE, PS, 5-methyl cytosine	Splicing modulation	Intron 6 spice acceptor cryptic site/CNS	CLN7	ITH/42 mg once every 3 months	FDA (2018)	Orphan
Volanesorsen (Waylivra^®^), Ionis Pharma, Akcea Pharma	20 mer, PS,2′-O-MOE	RNAse H	mRNA APOCIII/liver	FCS	SC/285 mg once weekly	EMA (2019)	Orphan
Givosiran (Givlaari^®^), Alnylam	PS - SiRNA GalNAc	AGO2	ALAS1 mRNA/liver	AHP	SC/2,5 mg/kg once every months	FDA (2019),	Orphan
						EMA (2020)	
Golodirsen (Vyondys 53™), Sarepta Tx	25 mer PMO	Exon-Skipping	DMD pre-mRNA/muscle	DMD	I.V/30 mg/kg once weekly	FDA (2019)	Orphan
Viltolanersen (Viltepso™), NS Pharma	PMO	Exon-skipping	DMD pre-mRNA/muscle	DMD	IV/80 mg/kg once weekly	FDA (2020)	Orphan
Lumasiran (Oxlumo™), Alnylam	siRNA	AGO2	HA01 mRNA/liver	PH1	SC/dose and frequency depend on the patient’s weight	FDA (2020)	Orphan
						EMA (2020)	
Inclisiran (Leqvio^®^), The Medicines Company, Novartis	2′F, 2′-O-Me, PS siRNA- GalNAc	AGO2	mRNA PCSK9/liver	FH	SC/300 mg once every 6 months	EMA (2020)	-
						FDA (2021)	
Casimersen (Amondys 45 ^TM^), Sarepta	22 mer PMO	Exon-Skipping	DMD pre-mRNA/muscle	DMD	30 mg/kg once weekly	FDA (2021)	Orphan
Tofersen	2′-O-MOE-PS	RNase H1	SOD1 mRNA/brain	ALS	100mg/15 mL (6.7 mg/mL) single-dose vial	FDA (2023)	

Antisense medicines approved by the Food and Drug Administration (FDA) or European Medicines Agency (EMA). 2′- F— 2′-Fluoro, 2′-O- MOE—2′-O-methoxyethyl, AHP—acute hepatic porphyria, ALAS—Aminolevulinate synthase, Apo—Apolipoprotein, CLN7— Neuronal Ceroid Lipofuscinosis, CMV—cytomegalovirus, CNS—central nervous system, DMD—Duchenne muscular dystrophy, FCS—Familial chylomicronemia syndrome, FH—Familial hypercholesterolemia, GAlNAc—N-acetylgalactosamine, HAO1— Hydroxyacid oxidase 1, hATTR—hereditary transthyretin amyloidosis, HoFH—omozygous familial hypercolesterolaemia, ITH—Intrathecal, IV—Intravenous, IVT—intravitreal, PCSK9—Proprotein convertase subtilisin/kexin type 9, PH1 — Hyperoxaluria type 1, PMO, Phosphorodiamidate morpholino; PS, Phosphorothioate, SC—Subcutaneous, siRNA—Small interfering RNA, SMA—spinal muscular atrophy, SMN—survival of motor neurons, TTR—Transthyretin.

### 6.1 Infectious diseases

#### 6.1.1 Fomivirsen

The first oligonucleotide available for therapy was fomivirsen, a phosphorothioate ASO approved by the FDA and EMA respectively in 1998 and 1999 for the second-line treatment of cytomegalovirus (CMV) retinitis in patients with acquired immunodeficiency syndrome (AIDS) (Geary et al., 2002). Fomivirsen, administered locally by intravitreal injection, had a complementary structure to the viral messenger RNA encoding CMV immediate-early (IE)-2 protein, required for viral replication (Geary et al., 2002). Clinical efficiency was demonstrated in a randomised clinical trial, in which fomivirsen slowed the progression of the disease over 71 days compared to 13 days in the control group (Hutcherson and Lanz, 2002). The progression of the disease happened in 44% of treated patients compared to 70% in untreated patients (Hutcherson and Lanz, 2002). Although a milestone in the field of ASOs, this treatment was used for a limited period, as the incidence of cytomegalovirus retinitis was dramatically reduced by the development of highly active antiretroviral therapy (HAART) (Yin and Rogge, 2019). Developed by Ionis Pharmaceuticals and subsequently licensed to Novartis, it was revoked in 2002 in the European Union and in 2006 in the United States (Stein and Castanotto, 2017).

### 6.2 Cardiovascular diseases

#### 6.2.1 Mipomersen

The first systemically approved ASO therapy was mipomersen, an antisense 20-mer phosphorothioate 2′-methoxy-ethoxy (MOE) gapmer, developed by Genzyme for adult patients with homozygous familial hypercholesterolaemia (HoFH) (Thomas et al., 2013). HoFH is an autosomal dominant genetic disorder characterised by high levels of low-density lipoprotein cholesterol (LDL-C) with a risk of developing coronary heart disease by the age of 30 (Cuchel et al., 2014). The disease is characterized by loss of function mutations in both LDL-receptor genes, causing the reduced liver uptake of plasma LDL cholesterol. Mipomersen forms a heteroduplex with a complementary mRNA sequence encoding for apolipoprotein B, the main component of LDL-C produced by the liver, resulting in the activation of RNase H (Akdim et al., 2010). The decreased production of apolipoprotein B consequently reduces the export of LDL-C from the liver, preventing the atherosclerotic process (Akdim et al., 2010). It is administered by subcutaneous injection at a dose of 200 mg once a week in addition to the maximum dose of hypolipidemic therapy and diet. Mipomersen was approved by the FDA in January 2013 on the basis of results obtained in four phase III clinical studies (Stein and Castanotto, 2017) in which mipomersen reduced plasma C-LDL concentration by 25%–37% compared to baseline (primary endpoint) in patients with HoFH already treated with a hypolipidemic drug (Hair et al., 2013). There were also reductions in total cholesterol, apolipoprotein B and lipoprotein (a) (Hair et al., 2013). In contrast, the EMA denied the marketing authorisation on 13 December 2012 due to concerns about side effects of a drug intended for long-term use, including liver toxicity and cardiovascular risk (Il GEB, 2013). Mipomersen did not accomplish a successful marketing as it was outperformed for minor hepatotoxic effects by a small molecule (lopitamide) (Stein and Castanotto, 2017). Its uncertain future is also linked to the competition of PCSK9 (Proprotein convertase subtilisin/kexin type 9) inhibitors, monoclonal antibodies (e.g., evolocumab and alirocumab) and oligonucleotide molecules recently approved (inclisiran) (Stein and Castanotto, 2017).

#### 6.2.2 Volanesorsen

Volesorsen is a PS, 2′-O-MOE gapmer, approved by the EMA in May 2019, suitable for use in adults affected by familial chylomicronemia syndrome (FCS), a rare genetic disorder with high risk of pancreatitis, in case the response to diet and the therapeutic lowering of triglycerides is insufficient (Paik and Duggan, 2019). Volanesorsen, inhibits hepatic apolipoprotein CIII (APOCIII) mRNA, resulting in reduced plasma apolipoprotein C-III levels (Paik and Duggan, 2019). Also the inhibition of APOC3, which encodes a protein involved in triglyceride (TG)-rich lipoproteins (TGRLs) removal, has been reported to be an original target to treat severe hypertriglyceridemia (sHTG). It is administered by subcutaneous injection at a dose of 285 mg once a week for the first 3 months of treatment and then once every 2 weeks. The frequency of administration is adjusted again after six and 9 months according to the patient’s clinical condition. Thrombocytopenia, observed in clinical trials, should be monitored in patients receiving the medicine (Witztum et al., 2019). The approval followed the results of the multinational phase III APPROACH study in which patients treated with volanesorsen showed a 77% reduction in average triglyceride levels and a significant reduction in pancreatitis attacks (Witztum et al., 2019). Additional clinical trials are ongoing to evaluate its efficacy in hypertriglyceridemia, familial chylomicronemia syndrome (FCS) and partial lipodystrophy (Paik and Duggan, 2019).

#### 6.2.3 Defibrotide

Defibrotide is approved by EMA and by FDA, respectively in 2013 and in 2016, for the treatment of patients with hepatic veno-occlusive disease (VOD) that occurs after high dose chemotherapy and autologous bone marrow transplantation (HSCT) (Stein and Castanotto, 2017). Liver veno-occlusive disease results from damage and occlusion of small hepatic venules due to endothelial cells activity triggered by local release of cytokines as part of pro-inflammatory and pro-thrombotic states and activation of the fibrinolytic pathway (Dalle and Giralt, 2016). Hepatic sinusoids become fibrous with necrosis of perivascular hepatocytes. Patients may develop jaundice, painful hepatomegaly, fluid retention, ascites and weight gain with progressive organ failure (up to 80% mortality) (Dalle and Giralt, 2016). Defibrotide is a mixture containing femtomolar concentration of single- (90%) and double-stranded (10%) ASOs derived from porcine intestinal mucosal DNA (Kornblum et al., 2006). The concentration of any specific sequence in the mixture is approximately in the femtomolar range. Therefore, defibrotide cannot act through an antisense mechanism but, most likely, through charge-charge interactions of its phosphodiester constituents with proteins (Stein and Castanotto, 2017). Although the mechanism of action is uncertain, defibrotide has antithrombotic and anti-inflammatory properties. Defibrotide is in fact able to increase the expression of systemic tissue factor pathway inhibitor (TFPI), tissue plasminogen activator (t-PA) and thrombomodulin (TM); reducing expression of von Willebrand factor (vWF) and plasminogen activator inhibitor-1 (PAI-1); and enhancing plasmin enzyme activity to promote hydrolysis of fibrin clots (Richardson et al., 2017). The recommended dose is 6.25 mg/kg body weight every 6 h (25 mg/kg/day) administered by intravenous infusion for at least 21 days and is to be continued until symptoms and signs of severe VOD have resolved (Stein and Castanotto, 2017). The study had two endpoints: the primary one was the patient survival rate at day +100 post-HSCT. The percentage of patients alive in the DF group was 38.2% (39/102) versus 25% (8/32) in the control group. The secondary one was the complete response rate (defined as total bilirubin levels below 2 mg/dL), with a rate of 23.5% (24/102) observed in the DF-treated group and a rate of 9.4% (3/32) in the control group (Richardson et al., 2016).

#### 6.2.4 Inclisiran

Inclisiran is a 2′F, 2′-O-Me, PS siRNA-GalNAc, approved by both the EMA and FDA on the basis of surrogate endpoints for the use in adults with primary hypercholesterolaemia or mixed dysplidaemia as a supplement to standard diet and therapy (Food and Drug Admi nistration, 2020). These diseases result in elevated blood cholesterol levels with a predisposition to atherosclerotic disease.

Inclisiran should be used in combination in patients who, with the maximum tolerated dose of statins, are unable to reach low-density lipoprotein cholesterol goals. Inclisiran, by activating AGO2, induces cleavage of the mRNA encoding for proprotein convertase subtilisin-kexin type 9 (PCSK9), an enzyme that negatively regulates the turnover and therefore LDL receptor (LDLR) levels on the hepatocyte membrane (Dyrbuś et al., 2020). This results in increased uptake of LDL from the bloodstream due to increased expression of LDLR levels (Dyrbuś et al., 2020). Chemical modifications and conjugation with three N-acetyl-galactosamine molecules significantly improve the biodistribution of the drug, allowing the cholesterol-lowering effect to be maintained for up to 6 months with a single subcutaneous injection of the medicine (Fitzgerald et al., 2017). Evidence supporting the efficacy of inclisiran comes from randomised, placebo-controlled phase III clinical trials. Among these studies, ORION-10 and ORION-11 were the studies with the highest number of patients (Dyrbuś et al., 2020). ORION-10 tested the medicine in 1,561 patients with atherosclerotic cardiovascular disease (ASCVD) and ORION-11 included 1,617 subjects with ASCVD or equivalent cardiovascular risk. At the end of the follow-up period (day 510), inclisiran reduced LDL-C levels by 52.3% in ORION-10% and 49.9% in ORION-11 (Dyrbuś et al., 2020). Currently, the ORION-4 study is underway to examine the impact of inclisiran (in addition to standard therapy and diet) in reducing the risk of major adverse cardiovascular events in approximately 15,000 ASCVD patients.

### 6.3 Metabolic and endocrine diseases

#### 6.3.1 Givosiran

Givosiran is a GalNAc-conjugated PS-siRNA used to treat acute hepatic porphyria (AHP) in adults and adolescents over 12 years of age (Scott, 2020). Givosiran binds a ligand that enable the specific delivery of the siRNA to the liver, where it acts against the aminolevulinate synthase 1 (ALAS1) mRNA, resulting in decreased levels of neurotoxic δ-aminolevulinic acid and porphobilinogen, that are associated with acute porphyria attacks (Balwani et al., 2020). Administered by subcutaneous injection at a dose of 2.5 mg/kg once a month, givosiran enters the hepatocytes and degrades the mRNA encoding for ALAS1 by activation of AGO 2 (Scott, 2020). Approved by the FDA in 2019 and by the EMA in 2020, the efficacy of givosiran was evaluated in a randomised, double-blind, placebo-controlled clinical trial in a 1:1 ratio (ENVISION NCT03338816) (Balwani et al., 2020). Out of a total of 94 patients with AHP, 74% of patients treated with givosiran showed a reduction in porphyria attacks. Givosiran was well tolerated with common side effects such as nausea and injection site reactions (Balwani et al., 2020). Liver function and allergic reactions should be monitored during and after treatment (Dhuri et al., 2020).

#### 6.3.2 Lumasiran

Developed by Alnylam Pharmaceuticals and approved in November 2020 by both the FDA and the EMA, lumasiran is a subcutaneously administered siRNA specifically to reduce hepatic overproduction of oxalate in patient with primary hyperoxaluria type 1 (PH1) (Scott and Keam, 2021). A high level of oxalate in the urine can cause kidney stones or kidney failure (Frishberg et al., 2019). By targeting the hydroxyacid oxidase 1 (HAO1) mRNA in the liver, lumasiran decrease the production of glycolate oxidase. The reduced production of this enzyme results in an increase in the concentration of the precursor glycolate, which being soluble, is readily eliminated by the kidney (Scott and Keam, 2021). The drug dose is based on body weight. The clinical efficacy of lumasiran was evaluated in a randomized, double-blind trial comparison study between lumasiran and placebo in 39 patients aged 6 and 60 years with primary hyperoxaluria type 1. After 6 months of treatment, the level of oxalate in urine was reduced by 65% on average in patients on lumasiran compared with 12% in patients who received placebo (Garrelfs et al., 2021).

### 6.4 Neurological and neuromuscular diseases

#### 6.4.1 Nusinersen

Nusinersen is an antisense 18-mer phosphorothioate 2′-methoxy-ethoxy (MOE) gapmer engineered to treat patients with Spinal muscular atrophy 5q (SMA), an autosomal recessive degenerative motor neuron disease (Chiriboga, 2017). This disease is caused by a deletion or mutation with total loss of function of the survival motor neuron 1 (SMN1) on chromosome 5q. SMN protein deficiency causes weakness and atrophy of the limbs and respiratory and bulbar muscles (Chiriboga, 2017). Typically, humans have SMN2, a paralogous copy of the SMN1 gene. SMN2 differs from SMN1 by a basic cytosine to thymine transition in exon 7 that results in a weakened splice site. Consequently, 90% of the transcripts derived from SMN2 induce the splicing of this exon resulting in a truncated protein that is rapidly degraded.

ASO-based approaches aimed at exon 7 splicing correction (Hua et al., 2008) have employed several targets including the 3′ splice site (3′ ss) of exon 8, element 1, GC-rich sequence (GCRS), ISTL1 and ISS-N2 and the 100th position of intron 7. Among these, the largest number of studies has been done on Intronic Splice Silencing site (ISS-N1)-targeting ASOs (Singh et al., 2015).

Nusinersen increases the inclusion rate of exon 7 in SMN2 gene transcripts by binding to ISS-N1 and displacing splicing factors by steric hindrance (Singh et al., 2006; Singh et al., 2017; Goodkey et al., 2018). It is administered intrathecally at a dose of 12 mg. Treatment with nusinersen involves four loading doses (12 mg) on days 0, 14, 28 and 63 and a maintenance dose administered every 4 months (Wurster et al., 2019).

Nusinersen was approved in December 2016 by FDA and in May 2017 by EMA on the basis of increased survival and improved motor function demonstrated in two randomised, double-blind, simulation-controlled phase III clinical trials (Finkel et al., 2017; Mercuri et al., 2018). The clinical benefit shown, allowed to stopped early the placebo-controlled studies in order to generate an open-label study to receive the treatment (Yin and Rogge, 2019). Osredkar et al. recently have conducted a two-center study (in Slovenia and Czech Republic) which confirm the efficacy and safety of nusinersen after 14 months of treatment. The patient population under consideration included 61 subjects aged between 2 months and 19 years with a genetically confirmed diagnosis of SMA (Osredkar et al., 2021). Specifically, the results showed that all patients benefited from the drug. However, better outcomes were recorded with an earlier age of treatment initiation and a higher number of SMN2 copies. Neonatal screening may therefore be necessary to maximize drug efficacy. In terms of safety, no side effects made it necessary to stop treatment (Osredkar et al., 2021).

#### 6.4.2 Milasen

Milasen is an outstanding example of ASO-based personalised medicine as it is an ASO specifically designed for a girl suffering from neuronal ceroid lipofuscinosis 7 (CLN7), a disease that primarily affects the nervous system causing vision loss, dysarthria, ataxia and dysphagia (Kim et al., 2019). It is caused by an alteration in the transcript splicing due to the insertion of an SVA retrotransposon (SINE-VNTR-Alu) into the MFSD8 gene (also called CLN7). Milasen restores correct splicing by binding cryptic splice sites in the pre-mRNA (Kim et al., 2019). It is administered as an intrathecal bolus starting with 3.5 mg and increasing the dose every 2 weeks to 42 mg. After the dose increase, two additional loading doses are administered, followed by a maintenance dose approximately every 3 months (Kim et al., 2019). The safety of milasen has been assessed in preclinical toxicity studies (Dhuri et al., 2020).

#### 6.4.3 Patisiran

Transthyretin-mediated hereditary amyloidosis (hATTR) is caused by a mutation in the gene coding for transthyretin (TTR), a protein produced by the liver that transports thyroxine and retinol in the plasma and cerebrospinal fluid (Hanna, 2014). The result of the mutation is the formation of an oversized protein that accumulates as insoluble amyloid fibrils in the nerves, heart and gastrointestinal tract. In addition to polyneuropathy, this disease leads to a decline in cardiac function with a life expectancy of 3–15 years after the appearance of symptoms (Hanna, 2014). Patisiran, a 2′-O-Me, 2′F, PS siRNA formulated in a lipid nanoparticle, is directed at gene silencing of a transthyretin-coding mRNA sequence by activation of AGO2 (Hoy, 2018). The recommended medicine dose is 300 mg/kg body weight administered by intravenous infusion once every 3 weeks. Premedication with dexamethasone, acetaminophen/paracetamol and H1 and H2 antagonists is required to reduce the pro-inflammatory effect of the formulation (Adams et al., 2017). In 2018, it was approved by both FDA and EMA on the basis of robust evidence of clinical efficacy with an acceptable safety profile in patients with hereditary transthyretin-mediated amyloidosis with polyneuropath, demonstrated in a double-blind, randomised, placebo-controlled phase III study (APOLLO II; NCT01960348) (Hoy, 2018; Adams et al., 2017). Both the primary endpoint of the study (mNIS + 7, modified Neuropathy Impairment Score +7) and the secondary endpoints (quality of life, muscle strength, gait speed, nutritional status and autonomic function) were widely demonstrated (Adams et al., 2017). Compared to inotersen, there were no serious side effects. Three post-approval clinical studies are currently underway. The aim of the first study is to evaluate the long-term efficacy and safety of patisiran (expected end date August 2022); the second study evaluates the safety and efficacy of patisiran in patients with hATTR after liver transplantation; the third study compares the efficacy of vitrusiran with patisiran for the treatment of hATTR (Dhuri et al., 2020).

#### 6.4.4 Inotersen

Inotersen is a 2′-O-MOE PS gapmer ASO that has been approved by both the EMA and FDA in 2018 for the treatment of hATTR (Goodkey et al., 2018). Inotersen mediates the cleavage of mRNA encoding for transthyretin through the activation of RNAse H (Goodkey et al., 2018). It is administered by subcutaneous injection at a dosage of 284 mg once a week. The clinical efficacy of inotersen was demonstrated in a randomised, double-blind, placebo-controlled clinical trial (NEURO-TTR, NCT01737398) (Benson et al., 2018). However, there were two significant adverse reactions in the treatment group: thrombocytopenia and glomerulonephritis, which required the introduction of metabolic and haematological monitoring (Dhuri et al., 2020). Interesting are the results obtained in a study conducted to evaluate the long term safety and efficacy of inotersen in transthyretin cardiomyopathy (Dasgupta et al., 2020). As cardiomyopathy is a major cause of death in patients with systemic transthyretin amyloidosis, this results showed that inotersen is safe and effective in this context (Dasgupta et al., 2020).

#### 6.4.5 Eteplirsen

Duchenne muscular dystrophy (DMD) is an autosomal recessive myophathy caused by mutations in the dystrophin gene. Patients usually experience premature death around 30/40 years of age due to respiratory or cardiac failure (Aartsma-Rus and Krieg, 2017). Dystrophin deficiency occurs at the age of 2–5 years with delayed walking, hypotonic limbs, weakness and loss of muscle function. Gold-standards in the management of complications are corticosteroids and artificial respirators. Additionally, drug therapies including eteplirsen have been approved by the FDA to treat specific types of DMD. Eteplirsen is a 30-nucleotide PMO indicated for the treatment of DMD in patients with a specific genetic mutation that causes the deletion of exons 49–50, leading to the formation of a stop signal on codon 51. It is designed to bind to exon 51 of dystrophin pre-mRNA and promote the skipping of exon 51, excluding this exon from the primary dystrophin transcript (Vegeto et al., 2019). The final transcript results in a truncated but partially functional protein, as occurs in Becker muscular dystrophy (milder form of the dystrophic phenotype) (Beckers et al., 2021). It is administered by weekly venous infusion at a dose of 30 mg/kg. The clinical efficacy of eteplirsen was evaluated in a small group of patients in a phase II study in which the primary endpoints were the distance covered in 6 minutes and the percentage of dystrophin-positive fibres (Mendell et al., 2013). There were no significant differences in the gait test between groups and a moderate increase in dystrophin expression in muscle tissue was detected by immunohistochemical analysis (from 0.16% at baseline to 0.48% at week 48) (Mendell et al., 2013). The reasons for a weak physiological response to eteplirsen are due to a reduced absorption in muscle and a rapid renal filtration due to low plasma protein binding (Levin, 2019). In 2016 the FDA granted controversial accelerated approval based on surrogate endpoints (Mendell et al., 2013) to eteplirsen given the unsatisfied medical need, the severity and progressive nature of the disease. This authorization is subject to the completion of further studies confirming the function benefit from eteplirsen treatment. Otherwise, in 2018, the EMA refused the marketing of the medicine due to lack of data about the clinical benefit (Aartsma-Rus and Goemans, 2019).

#### 6.4.6 Golodirsen

Golodirsen is a ASO with 25 monomers suitable for the treatment of DMD (Heo, 2020). Similar to eteplirsen, it has a neutral synthetic backbone (PMO chemistry), however, it is targeted at patients with a different genetic mutation. Specifically, it is recommended for the treatment of DMD in subjects with a confirmed mutation in the dystrophin gene susceptible to skipping exon 53 (approximately 8% of DMD patients). Golodirsen is engineered to make exon 53 skip in the dystrophin gene, enabling the production of a truncated but still functional form of dystrophin, which is a deficient protein in the disease (Heo, 2020). The recommended dose of golodirsen is 30 mg/kg, administered by intravenous infusion (Heo, 2020). Renal function should be monitored in patients taking the medicine, as renal toxicity has been observed during preclinical *in vivo* studies (Frank et al., 2020). Golodirsen was approved in December 2019 by the FDA following positive results from a phase I/II clinical trial, which demonstrated increased dystrophin expression at the muscle level (Frank et al., 2020). The approval is subject to the implementation of further clinical studies confirming drug efficacy, i.e., improvement in mobility function, measured as the change in the 6-min walk test (6MWT) from baseline to week 26. The multicentre, randomised, double-blind, placebo-controlled phase III ESSENCE study is currently taking place and is expected to be completed by 2024. This study aims at assessing not only the safety and efficacy of golodisen but also of casimersen, an oligonucleotide that was approved by FDA for DMD patients with a mutation in dystrophin exon 45.

#### 6.4.7 Viltolanersen

Viltolarsen, an antisense phosphorodiamidate morpholino oligonucleotide, is specific for exon 53 of the dystrophin mRNA precursor. It has the capability to induce exon 53 skipping, generating a functional truncated dystrophin protein in DMD patients with a specific mutation (Dhillon). It is administered intravenously once a week for 1 h at a dose of 80 mg/kg. Renal function monitoring is required for patients receiving viltolanersen. Viltolanersen received accelerated marketing approval from the FDA in August 2020 on the basis of a multicentre phase II clinical trial, which showed increases in *de novo* dystrophin production in muscle (surrogate endpoint) and clinical improvements in time-based functional tests (Clemens et al., 2020).

#### 6.4.8 Casimersen

Casimersen is an antisense phosphorodiamidate oligonucleotide designed for exon skipping in patients diagnosed with DMD confirmed by the susceptible exon 45 skip genotype (Shirley, 2021). This drug allows the increase of dystrophin production at the skeletal muscle level through the synthesis of an internally truncated but partially functional protein in patients with DMD.

Casimersen is administered by intravenous infusion at the dose of 30 mg/kg once weekly (Shirley, 2021).

In February 2021, Casimersen received accelerated approval from the FDA based on the results of the ongoing, double-blind, placebo-controlled phase 3 study. Intermediate results showed a greater change in dystrophin expression from baseline in the casimersen treated group compared to placebo controls.

## 7 Future perspectives

The number of ASO molecules under investigation for the treatment of several diseases is considerable. The main candidates in phase III clinical trials are briefly described below in Table 2. It should be noted that, to date, these molecules are still mainly intended for local delivery (to the eye by intravitreal injection, to the central nervous system by intrathecal administration, to the colon by enema); and for delivery to the liver and kidney, the major organs where ASOs accumulate. As documented by the most recently approved drugs, oligonucleotides can be used both in personalised medicine (e.g., milasen) and for the treatment of a significant population of individuals (e.g., inclisiran). In the context of optimizing the development of ASOs, it is important to discuss the critical aspects of their design. The size of ASOs appears to play a pivotal role in reducing off-target effects, and careful consideration should be given to this parameter. Moreover, more extensive RNA-Seq and proteomic studies are needed to gain a deeper understanding of off-target effects that cannot be reliably predicted by currently available bioinformatic algorithms. These studies provide detailed information about how proteins are produced and how genes are expressed at the RNA level. This information can be crucial for assessing their off-target effects and also ensuring the safety and efficacy of ASOs in clinical applications. Additionally, a key challenge in the advancement of ASO-based therapeutics is the identification of chemical structures able to ensure better pharmacokinetics and pharmacodynamic properties. While GAlNAc conjugates and encapsulation in lipid nanoparticles (LNPs) have demonstrated excellent delivery to the liver, particularly for metabolic liver-related diseases, further research and advancements are required to achieve meaningful and widespread delivery to other tissues. Tissue-specific targeting represents a promising avenue for achieving pharmacological effect at lower doses and, at the same time, decrease the main causes of unresolved toxicity, including off-target accumulation in renal and hepatic tissues and proinflammatory effects.

**TABLE 2:** Investigational antisense drugs.

Drug candidate NCT ID	Chemistry	Mechanism of action	Target/organ	Indication	Route	Company
Tominersen (RG6042)	2′-O-MOE-PS	RNase H1	HTT mRNA/brain	Huntington’s disease	ITH	Ionis
Casimersen	PMO	Exon skipping	DMD exon 45/muscle	DMD	IV	Sarepta
AKCEA-TTR-LRx 04136171	siRNA GalNAc	RNAi	TTR mRNA/liver	ATTR with cardiomyopathy	SC	Ionis
QPI-1002	siRNA	RNAi	p53 mRNA/kidney	Cardiac surgery/kidney delayed graft function/acute kidney injury	IV	Quark
Fitusiran/ALN-AT3	siRNA GalNAc	RNAi	Anti-thrombin mRNA/liver	Haemophilia A and B	SC	Genzyme
Volanesorsen	2′-O-MOE-PS	RNase H1	ApoC-III mRN/liver	FCS/Lipoprotein Lipase Deficiency/Hyperlipo proteinemia type 1	SC	Ionis
Alicaforsen	PS	RNase H1	ICAM1 mRNA/colon	Pouchitis	E	Atlantic
Tivanisiran	siRNA	RNAi	TRPV1/eye	Dry eye syndrome	IVT	Sylentis
IONIS-TTR Rx	2′-O-MOE-PS	RNAse H1	TTR mRNA/liver	FAP/CS	SC	Ionis

Antisense drugs that showed promise in phase III, clinical trials 2′-O- MOE—2′-O-methoxyethyl, ALS—amyotrophic lateral sclerosis, ATTR—Transthyretin amyloidosis, DMD—Duchenne muscular dystrophy, E—Enema, Apo—Apolipoprotein, FAP—familial amyloid poly neuropathy, FCS—Familial chylomicronemia syndrome, GAlNAc—N-acetylgalactosamine, HTT—Huntingtin, ICAM—Intercellular adhesion molecule, ITH—Intrathecal, IV—Intravenous, IVT—intravitreal, PMO, Phosphorodiamidate morpholino; PS, Phosphorothioate, SC—Subcutaneous, siRNA—Small interfering RNA, SOD1 —Superoxide dismutase, TTR—Transthyretin, p53—Tumor protein, TRPV1—Transient Receptor Potential Vanilloid 1.

## 8 Conclusion

Major chemical and technological improvements have been made to diminish the toxicity and increase the efficacy of ASOs. This process has recently led to a great number of ASO-based medicines being used in clinical practice and to a renewed interest from big pharma, resulting in a multibillion-dollar market, comparable to that of small molecules. ASOs may be used in monotherapy or in combination with traditional therapy. They constitute a valid therapeutic alternative for diseases with no cure or effective therapies (orphan genetic diseases).

Thanks to their properties, ASOs are able to regulate oxidative stress and inflammatory response that are common mechanisms altered in many disorders. Currently, ASOs are primarily intended for the treatment of neurological, neuromuscular and metabolic disorders. However, thanks to further technological innovations, ASOs are expected to be used as advanced therapeutics for many other disorders in the next future.

## Figures and Tables

**Table:** 

**2′- F**	2′-Fluoro
**2′-O-Me**	2′-O-methyl
**2′-O-MOE**	2′-O-methoxyethyl
**6 MWT**	6 min walk test
**AGO-2**	Argonaut 2
**AHP**	Acute hepatic porphyria
**AIDS**	Acquired immunodeficiency syndrome
**ALAS**	Aminolevulinate synthase
**ALS**	amyotrophic lateral sclerosis
**aODN**	antisense OligoDesoxiNucleotides
**APO**	Apolipoprotein
**ATTR**	Transthyretin amyloidosis
**ASCVD**	Atherosclerotic cardiovascular disease
**ASGR**	Asialoglycoprotein receptor
**ASO**	Antisense Oligonucleotides
**BNA**	Bridged nucleic acid
**CLN7**	Neuronal Ceroid Lipofuscinosis
**CMV**	Cytomegalovirus
**CNS**	Central nervous system
**CPP**	Cell-penetrating peptides
**CRISP**	Clustered Regularly Interspaced Short Palindromic Repeats
**crRNA**	CRISP RNA, DNADeoxyriboNucleic Acid
**DF**	Defibrotide
**DMD**	Duchenne muscular dystrophy
**EMA**	European Medicines Agency
**E**	Enema
**EPR**	Enhanced permeability and retention
**FAP**	Familial amyloid poly Neuropathy
**FCS**	Familial chylomicronemia syndrome
**FDA**	Food and Drug Administration
**FH**	Familial hypercholesterolemia
**GAlNAc**	N-acetylgalactosamine
**HAART**	Highly active antiretroviral therapy
**HAO1**	Hydroxyacid oxidase 1
**hATTR**	Hereditary Transthyretin amyloidosis
**HoF**	HOmozygous familial hypercolesterolaemia
**HSCT**	Hematopoietic stem cell transplantation, HTTHuntingtin
**ICAM**	Intercellular adhesion molecule
**IE2**	Immediate early 2
**ITH**	Intrathecal
**IV**	Intravenous
**IVT**	intravitreal
**LDL-C**	Low-density lipoprotein cholesterol
**LDL-R**	Low-density lipoprotein receptor
**LNP**	lipid nanoparticles
**mRNA**	RNA messenger
**MFSD8**	Major Facilitator superfamily domain containing 8
**miRNA**	microRNA
**mNIS+7**	modified neuropathy impairment score +7
**p53**	Tumor protein, PAI—plasminogen activator inhibitor
**PBAE**	poly-(beta-amino-ester)
**PCSK9**	Proprotein convertase subtilisin/kexin type 9
**PEG**	Polyethylene glycol
**PEI**	Polyethylenimine
**PH1**	Hyperoxaluria type 1
**PMO**	Phosphorodiamidate morpholino
**PNA**	Nucleic peptide acid
**pre-mRNA**	precursor RNA messenger
**PS**	Phosphorothioate
**QOL-DN**	Norfolk Quality of Life-Diabetic Neuropathy
**RISC**	RNA Induced Silencing RNA
**RNA**	RiboNucleic Acid
**RNAi**	RNA interference
**RNAse H**	ribonuclease H
**SC**	Subcutaneous
**SELEX**	Systematic Evolution of Ligands by Exponential Enrichment
**sHTG**	severe hypertriglyceridemia
**siRNA**	Small interfering RNA
**SMA**	Spinal muscular atrophy
**SMN**	Survival of motor neuron
**SNA**	Spherical nucleic Acid
**SOD1**	Superoxide dismutase
**SOS**	Sinusoidal obstruction syndrome
**STAB**	stabilin
**SVA**	SINE-VNTR-Alu
**TG**	Triglyceride
**TGRL**	Triglyceride rich lipoprotein
**TFPI**	Tissue factor pathway inhibitor
**TLR**	Toll-like receptor
**TM**	thrombomodulin
**tPA**	tissue plasminogen activator
**TRBP**	TAR RNA binding protein
**TRPV1**	Transient Receptor Potential Vanilloid 1
**TTR**	Transthyretin
**VEGF**	vascular endothelial growth factor
**VOD**	Veno-occlusive disease
**vWF**	von Willebrand factor
